# Sex-Specific Differences in Peripheral Nerve Properties: A Comparative Analysis of Conduction Velocity and Cross-Sectional Area in Upper and Lower Limbs

**DOI:** 10.3390/diagnostics14232711

**Published:** 2024-11-30

**Authors:** Ayaka Nobue, Masaki Ishikawa

**Affiliations:** 1 Faculty of Medical Science Technology, Morinomiya University of Medical Sciences, Osaka 559-8611, Japan; 2 Graduate School of Sport and Exercise Sciences, Osaka University of Health and Sport Sciences, Osaka 590-0459, Japan

**Keywords:** nerve conduction velocity, sex, gender, ultrasonography, peripheral nervous system

## Abstract

**Background/Objectives:** Peripheral nerve conduction velocity (NCV) and nerve cross-sectional area (nCSA) are crucial parameters in neurophysiological assessments, yet their sex-specific differences are not fully understood. This study investigated sex-based variations in NCV and nCSA between upper and lower limbs. **Methods:** Twenty participants (ten males and ten females) were recruited for this study. The NCV and nCSA of the ulnar and tibial nerves were measured in both the upper and lower limbs. NCV was measured using supramaximal electric stimulation, and nCSA was assessed using peripheral nerve ultrasonography at three regions for each nerve. Supramaximal electric stimulations were applied superficially to the ulnar and tibial nerves at each measurement point. Action potentials were recorded from the abductor digiti minimi and soleus muscles for the ulnar and tibial nerves, respectively. **Results:** The ulnar nCSA of the upper limbs was significantly greater in males than in females (*p* < 0.05). However, ulnar NCV was significantly higher in females than in males (*p* < 0.05). In the lower limbs, no sex differences were observed in tibial NCV or nCSA. **Conclusions:** These findings reveal sex-specific differences in upper limb peripheral nerve characteristics that may have important implications for clinical assessments and treatment strategies. The contrasting patterns between upper and lower limbs suggest that both developmental and functional factors influence peripheral nerve properties.

## 1. Introduction

Peripheral nerve conduction velocity (NCV), the speed at which electrical signals propagate through peripheral nerves, is a fundamental physiological parameter that critically influences neuromuscular function and motor control. This parameter serves as a key indicator of peripheral nerve health and function, with significant implications for clinical diagnosis and treatment. Understanding sex-based differences in these parameters is particularly crucial as emerging evidence suggests that males and females may require different diagnostic criteria and treatment strategies.

Research over the past several decades has revealed complex relationships between NCV and various physiological and anatomical factors. Studies on lateral dominance have demonstrated that NCV is higher in the dominant hand than in the non-dominant hand [1]. Similarly, investigations involving athletes have shown elevated NCV in the dominant arms of badminton players and baseball pitchers relative to non-athletes [2,3,4,5]. These findings initially suggested a potential relationship between physical development and NCV, with some studies reporting higher ulnar NCV in males compared to females [6]. However, subsequent investigations have revealed more complex patterns, particularly regarding sex differences. Studies comparing males and females have shown that males with larger arm muscles exhibit lower NCV than females with smaller arm muscles [7,8]. This paradoxical relationship indicates that limb size may not be a reliable predictor of NCV in humans, highlighting the need for a more comprehensive and multifaceted analysis of the factors influencing NCV, particularly regarding sex-based differences.

Recent advances in neuroscience have demonstrated significant sex-based differences in the structure and function of the central nervous system (CNS) [9]. For example, females have more intricate brain folding patterns, a higher proportion of white matter, and sex-specific brain circuitry. These differences persist even when controlling for body size and suggest fundamental biological variations in neural organization between males and females [10,11]. Given these well-documented sex-based differences in the CNS, our understanding of sex-based differences in peripheral nerve characteristics remains limited, particularly regarding the relationship between nerve structure and function.

When exploring potential sex-based differences in the peripheral nervous system, it is important to consider the fundamental properties of nerve conduction. Animal studies have shown that NCV increases with the thickness of nerve fibers [12,13,14], indicating that morphological characteristics play a significant role in determining NCV. In the peripheral nerve morphology, Magnaghi et al. [15] reported the sex differences of progesterone and its metabolites, which influence the gene expression of myelin proteins in Schwann cells and suggested sex-specificity in the shape of peripheral nerves. In humans, nerve fibers cannot be directly identified in vivo. Therefore, it is still unclear whether functional differences in peripheral nerves are attributable to morphological variations, sex-specific factors, or environmentally induced alterations between the sexes.

Recent advancements in peripheral nerve ultrasonography have made it possible to identify the nerve trunk level, which consists of bundles of nerve fibers, e.g., Refs. [16,17,18,19]. Using this technique, previous research has revealed unique characteristics of nerve structure and function. For example, Nobue et al. [19] found an interesting dissociation between nerve cross-sectional area (nCSA) and NCV in the lower limbs; the reacting leg exhibited higher NCV compared to the supporting leg despite having a smaller nCSA and limb circumference. This finding suggests potential differences in how peripheral nerve properties may be influenced by functional specialization between limbs. Given that upper and lower limbs serve different functional roles and experience different patterns of use, we hypothesized that sex-specific characteristics in peripheral nerves might also show limb-specific patterns. To test this hypothesis, the purpose of this study was to examine sex-specific differences in human NCV and nCSA between the upper and lower limbs.

## 2. Materials and Methods

### 2.1. Participants

Participants were recruited through university advertisements and screened for eligibility using a health questionnaire. Twenty healthy volunteers with no history of neurological disorders, peripheral neuropathy, or upper limb disorders, and no bilateral differences in forearm length participated in this study [10 males and 10 females; Age: male 20.3 ± 2.2 years, female 20.1 ± 1.6 years; Body mass: male 72.7 ± 8.5 kg, female 55.9 ± 6.0 kg, Height: male 175.1 ± 7.6 cm, female 160.7 ± 6.0 cm]. Exclusion criteria included any history of peripheral nerve injury, diabetes, or neuromuscular disorders. The Edinburgh Handedness Inventory [20] was used to determine hand dominance, while the Waterloo Footedness Questionnaire [21] was employed to identify the dominant (reacting) and non-dominant (supporting) legs. All participants provided informed consent prior to the experiment. This study was conducted in accordance with the guidelines of the Declaration of Helsinki and was approved by the Ethics Committee of the Osaka University of Health and Sport Sciences (authorization number 19-8).

### 2.2. Protocols

We selected the ulnar and tibial nerves based on several key methodological advantages. These nerves provide consistent and reliable accessibility for both ultrasonographic imaging and electrical stimulation, primarily due to their relatively superficial anatomical locations and well-defined anatomical landmarks [19,22]. Moreover, they demonstrate minimal anatomical variation compared to other major peripheral nerves, which reduces potential confounding factors in cross-sectional measurements. From a functional perspective, these nerves serve comparable motor and sensory roles in the upper and lower limbs, making them ideal for our comparative study design. Their anatomical courses include multiple suitable points for stimulation, enabling precise measurement of conduction velocities over sufficient distances. Previous studies have demonstrated high measurement reliability for both NCV and ultrasonographic assessments of these nerves [17,18,23,24].

The experimental protocol consisted of comprehensive neuromuscular assessments using three standardized measurement techniques performed under controlled laboratory conditions (ambient temperature: 22 ± 1 °C). First, we measured limb circumferences at standardized anatomical locations using a flexible, non-elastic measuring tape calibrated to 0.1 cm precision. For upper limbs, measurements were taken at the maximum forearm girth with the elbow extended, while lower limb measurements were obtained at the maximum calf circumference in a standing position. Next, peripheral nerve morphology was evaluated using high-resolution ultrasound imaging. The nCSA measurements were performed using an ultrasonographic system (Noblus, Hitachi Aloka Medical Ltd. Tokyo, Japan) with an 18 MHz linear array transducer providing 0.08 mm image resolution. During these assessments, participants maintained specific standardized positions: seated with 120° elbow flexion for ulnar nerve imaging and prone for tibial nerve examination. Finally, we assessed nerve conduction properties through electrophysiological recordings. NCV was determined using a constant current stimulator (DS7A, Digitimer Ltd., Welwyn Garden, UK) for supramaximal percutaneous stimulation, with muscle responses recorded via surface electrodes (P-EMG plus, Oisaka Electronic Equipment, Hiroshima, Japan). All measurements were performed bilaterally in randomized order to minimize systematic bias, with careful monitoring of skin temperature throughout the testing procedures.

### 2.3. Measured Parameters

#### 2.3.1. Nerve Cross-Sectional Area (nCSA)

**Figure 1:**
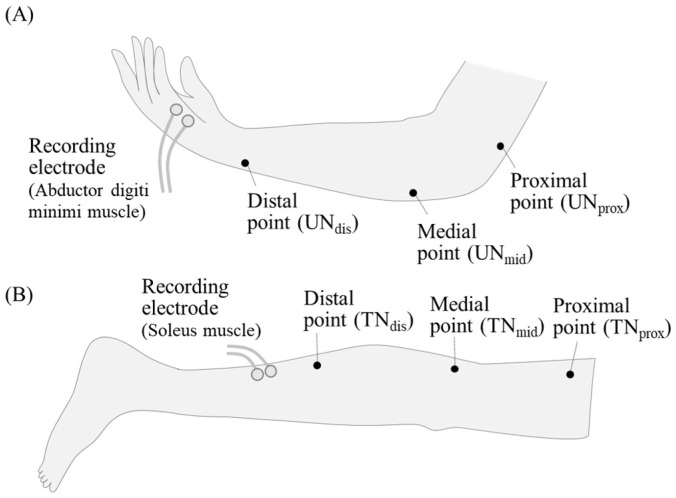
The measurement points of the cross-sectional area (CSA) and positions of nerve electrical stimulation of the ulnar and tibial nerves. (**A**) The measured positions of the circumstance, nCSA, and electrical stimulation for the ulnar nerve: UN_prox_, UN_mid,_ and UN_dis_. (**B**) The measured positions of the circumstance, nCSA, and electrical stimulation for the tibial nerve: TN_prox_, TN_mid_, and TN_dis_.

The ulnar and tibial nerves were scanned at three regions in the upper and lower limbs, respectively (Figure 1). In the upper limb, the first region was at 100 mm proximal point to the medial epicondyle of the humerus (UN_prox_), the second region was at 30 mm distal point to the medial epicondyle of the humerus (UN_mid_), and the third region was at 30 mm proximal point to the ulnar head (UN_dis_). In the tibial nerves, the first region was at 100 mm proximal point to the popliteal fossa (TN_prox_), the second region was at the popliteal fossa point (TN_mid_), and the third region was at 50 mm proximal point to the soleus muscle belly (TN_dis_). The nCSA, representing the bundle of nerve fibers, was measured using ultrasonographic images at each region of the ulnar and tibial nerves [22]. The nerve circumference boundary was traced on these images, and the nCSAs were analyzed separately for each point (UN_prox_, UN_mid_, and UN_dis_, TN_prox_, TN_mid_, and TN_dis_) using Image J software (version 1.45s, National Institutes of Health, Bethesda, MD, USA). The mean nCSAs for each limb were calculated by averaging the measurements from the three points.

#### 2.3.2. Upper and Lower Limb Circumferences

The circumferences of the upper and lower limbs were measured to provide context for the nCSA measurements. The circumference of the upper limb was measured around the maximal girth of the forearm. The circumference of the lower limb was measured around the maximal girth of the calf, typically at its widest point. All measurements were taken using a flexible, non-stretchable measuring tape, with the limb relaxed and in a neutral position. To ensure consistency, each measurement was taken three times, and the average was used for analysis. The cross-sectional area (CSA) for each arm and leg region was calculated from the respective circumference measurements using the following formula:

$$CSA=\frac{C^{2}}{4\pi }$$

where CSA = Cross-sectional area at each measurement point (cm^2^), *C* = Circumference at each measurement point (cm), and *π* = Pi (approximately 3.14159).

These limb CSA measurements provide a reference for comparing the relative size of the nerve to the overall limb size, which is important for interpreting the nCSA results in the context of individual body size variations.

#### 2.3.3. Motor Nerve Conduction Velocity (NCV)

NCV was measured using Signal software (Signal version 7.01, Cambridge Electronic Design Limited, Cambridge, UK) and an A/D converter (Power 1401, Cambridge Electronics Design Limited, UK). This system enabled high-fidelity signal capture at 10 kHz sampling frequency, ensuring accurate temporal resolution of nerve responses. Compound muscle action potentials (CMAPs) were evoked using an electrical stimulator (DS7A, Digitimer Ltd., Welwyn Garden, UK), delivering 0.2 ms duration constant current square wave pulses. Stimulation intensity progressed from minimal to supramaximal levels. For the ulnar nerve, the active electrode was placed on the belly of the abductor digiti minimi muscle, with a ground electrode on the ulnar head. Subjects were seated with their forearm flexed at 120°. For the tibial nerve, the active electrode was attached to the belly of the soleus muscle, with a ground electrode on the malleolus lateralis. These measurements were taken with subjects lying prone and their ankles in a neutral position. Stimulation points, identical to those used for nCSA measurements, were marked with an aqueous marker, and the distances between points were measured using a measuring tape. We determined nerve conduction parameters through systematic analysis of the recorded waveforms. The initial deflection of the M-wave from the baseline was precisely identified. NCV was quantified by analyzing the temporal displacement between proximal and distal response latencies, as described by Kimura [24]. Although NCV was measured separately in proximal and distal segments, we used the average of these measurements for analysis to account for anatomical variations in nerve diameter along its course. The mean values were calculated from UN_prox_ − UN_mid_ and UN_mid_ − UN_dis_ measurements for the ulnar nerve and TN_prox_ − TN_mid_ and TN_mid_ − TN_dis_ measurements for the tibial nerve. Throughout the measurements, skin and core body temperatures were monitored using a CORE device (greenTEG AG, Rümlang, Switzerland) to ensure consistency and avoid temperature-related influences on NCV.

### 2.4. Statistical Analyses

Results are presented as means ± standard deviations. Sex differences in physical characteristics were compared using two-tailed t-tests after confirming normal distribution with Shapiro–Wilk tests.

Analyses of upper and lower limb parameters incorporated both between-subject (sex) and within-subject (laterality) factors. Data were first examined using Mauchly’s sphericity test, followed by two-way repeated-measures ANOVAs (rmANOVAs). This approach enabled the assessment of the main effects and potential interactions between sex and limb dominance. Significant ANOVA results were further examined using Tukey’s post hoc tests to identify specific group differences. In cases where normality assumptions were not met, we applied Wilcoxon signed-rank tests with appropriate Bonferroni corrections.

Correlations between parameters were evaluated using Pearson’s correlation coefficients after confirming the normal distribution of variables. All statistical analyses were performed using Jamovi software (Version 2.3.28.0, The Jamovi Project 2024, Sydney, Australia).

To evaluate statistical power, post hoc analysis using G*Power software (version 3.1.9.7) indicated that our sample size achieved 0.56 power to detect medium effect sizes (Cohen’s *f* = 0.25) at α = 0.05. Effect sizes for rmANOVA were classified according to Cohen’s criteria [25] as small (*η_p_*^2^ = 0.01), medium (*η_p_*^2^ = 0.06), or large (*η_p_*^2^ = 0.14).

## 3. Results

### 3.1. Subject Characteristics

Table 1 presents the subject characteristics. Males were significantly taller [*t*_(18)_ = 4.708, *p* < 0.001, *d* = 2.105], heavier [*t*_(18)_ = 4.990, *p* < 0.001, *d* = 2.293], and had longer forearms and lower legs (all *p* < 0.05) compared to females.

### 3.2. Sex Differences in Measured Parameters

**Figure 2:**
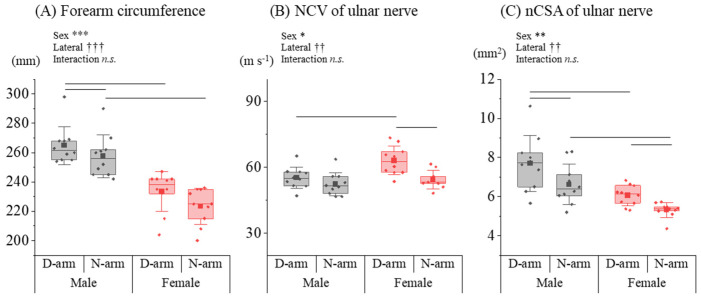
Sex differences in limb circumference, NCV, and nCSA for upper limbs. (**A**) The upper limb circumference, (**B**) the ulnar NCV, and (**C**) the ulnar nCSA for males and females were compared between the dominant and non-dominant arms (D-arm and N-arm). * and † indicate significant sex difference and lateral preference effect (* *p* < 0.05, ** *p* < 0.01, *** *p* < 0.001, †† *p* < 0.01, ††† *p* < 0.001, *n.s*.: not significant), respectively.

The analysis of upper limb parameters revealed distinct patterns based on sex. For the circumference of the forearm, the rmANOVA indicated significant main effects for sex [*F*_(1,18)_ = 39.722, *p* < 0.001, *η_p_*^2^ = 0.815] and lateral preference [*F*_(1,18)_ = 29.099, *p* < 0.001, *η_p_*^2^ = 0.764], with no interaction found [*F*_(1,18)_ = 1.213, *p* = 0.299, *η_p_*^2^ = 0.119] (Figure 2A). Post hoc Tukey’s tests showed that the forearm circumference of the male dominant arm was greater than both the male non-dominant arm (*p* = 0.012) and the female dominant arm (*p* < 0.001). Additionally, the male non-dominant arm circumference was significantly greater than that of the female non-dominant arm (*p* < 0.001). For ulnar NCV, significant main effects were also observed for sex [*F*_(1,18)_ = 6.139, *p* = 0.038, *η_p_*^2^ = 0.434] and lateral preference [*F*_(1,18)_ = 15.987, *p* = 0.004, *η_p_*^2^ = 0.666], with no interaction detected [*F*_(1,18)_ = 3.760, *p* = 0.088, *η_p_*^2^ = 0.320] (Figure 2B). Post hoc Tukey’s tests revealed that the ulnar NCV of the female dominant arm was significantly higher than that of the male dominant arm (*p* = 0.022) and the female non-dominant arm (*p* = 0.014). Regarding the ulnar nCSA, significant main effects of sex [*F*_(1,18)_ = 18.323, *p* = 0.002, *η_p_*^2^ = 0.671] and lateral preference [*F*_(1,18)_ = 16.276, *p* = 0.003, *η_p_*^2^ = 0.644] were found, with no interaction present [*F*_(1,18)_ = 0.452, *p* = 0.518, *η_p_*^2^ = 0.048] (Figure 2C). Post hoc Tukey’s tests showed that the ulnar nCSA of the male dominant arm was greater than that of the male non-dominant arm (*p* = 0.040) and the female dominant arm (*p* = 0.011). Furthermore, the female non-dominant arm had a significantly smaller ulnar nCSA compared to both the female dominant arm (*p* = 0.003) and the male non-dominant arm (*p* = 0.011).

**Figure 3:**
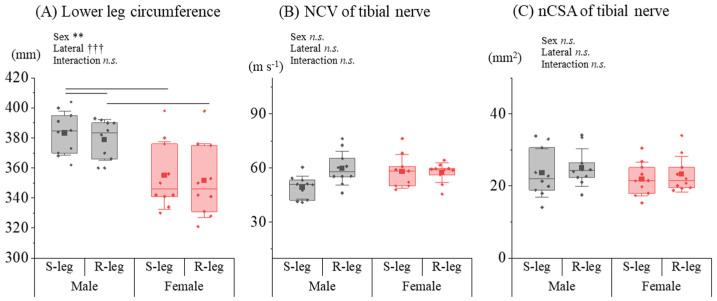
Sex differences in limb circumference, NCV, and nCSA for lower limbs. (**A**) The lower limb circumference, (**B**) the tibial NCV, and (**C**) the tibial nCSA for male and female groups were compared between the supporting and reacting legs (S-leg and R-leg), respectively. * and † indicate significant sex difference and lateral preference effect (** *p* < 0.01, ††† *p* < 0.001, *n.s*.: not significant), respectively.

The analysis of lower limb parameters revealed distinct patterns when compared to the upper limbs. Regarding the circumference of the lower leg, the rmANOVA indicated significant main effects for sex [*F*_(1,18)_ = 15.724, *p* = 0.003, *η_p_*^2^ = 0.636] and lateral preference [*F*_(1,18)_ = 44.301, *p* < 0.001, *η_p_*^2^ = 0.831]. There was no significant interaction found [*F*_(1,18)_ = 0.187, *p* = 0.676, *η_p_*^2^ = 0.020] (Figure 3A). Post hoc Tukey’s tests showed the lower leg circumference of the male supporting leg was significantly greater than that of the male reacting leg (*p* = 0.007) and the female supporting leg (*p* = 0.002). Additionally, the circumference of the male reaching leg was significantly larger compared to that of the female reaching leg (*p* = 0.006). For the tibial NCV, no significant main effects were found for sex [*F*_(1,18)_ = 2.918, *p* = 0.126, *η_p_*^2^ = 0.267] or lateral preference [*F*_(1,18)_ = 2.734, *p* = 0.137, *η_p_*^2^ = 0.255], nor was there any significant interaction [*F*_(1,18)_ = 3.354, *p* = 0.104, *η_p_*^2^ = 0.295] (Figure 3B). Regarding the tibial nCSA, none of the main effects were significant, including those for sex [*F*_(1,18)_ = 0.676, *p* = 0.442, *η_p_*^2^ = 0.070] and lateral preference [*F*_(1,18)_ = 1.200, *p* = 0.302, *η_p_*^2^ = 0.118]. There was also no significant interaction [*F*_(1,18)_ = 0.004, *p* = 0.953, *η_p_*^2^ < 0.001] (Figure 3C).

### 3.3. Comparison of Relative Values Between Males and Females

**Figure 4:**
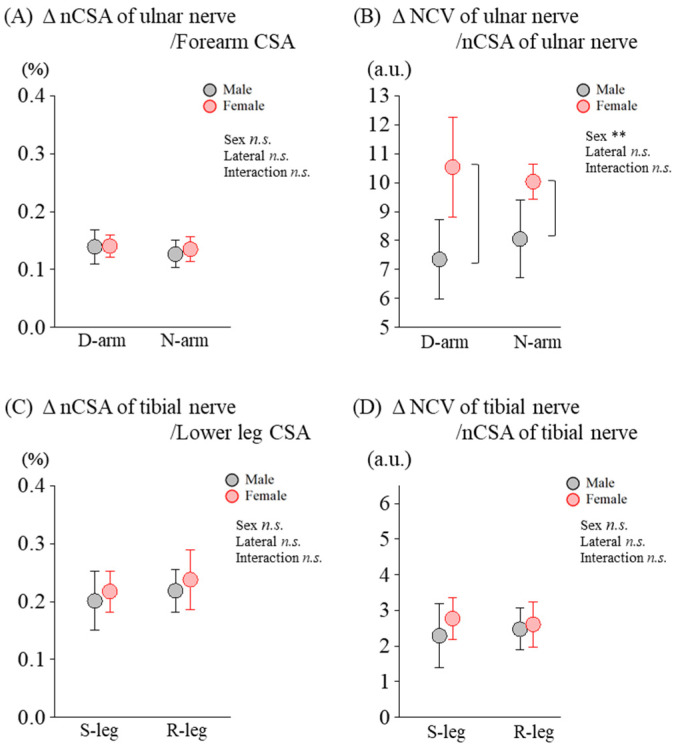
Sex differences in relative values for nerve conduction velocity (NCV) and nerve cross-sectional area (nCSA) in the upper and lower limbs. (**A**) Ratio of ulnar nCSA to upper limb CSA, (**B**) Ratio of ulnar NCV to ulnar nCSA, (**C**) Ratio of tibial nCSA to lower limb CSA, (**D**) Ratio of tibial NCV to tibial nCSA. Comparisons are made between dominant (D-arm) and non-dominant (N-arm) arms for upper limbs and between supporting (S-leg) and reacting (R-leg) legs for lower limbs. ** indicates a significant main effect of sex (*p* < 0.01). *n.s*. indicates no significant effects or interactions.

Given the observed sex differences and lateral dominance effects in normalized nCSA and NCV, we also examined relative values using the same analytical approach. For the ratio of ulnar nCSA to upper limb CSA, the rmANOVA revealed no significant main effects with sex [*F*_(1,18)_ = 0.303, *p* = 0.595, *η_p_*^2^ = 0.033] and lateral preference [*F*_(1,18)_ = 4.494, *p* = 0.063, *η_p_*^2^ = 0.333].There were also no significant interactions [*F*_(1,18)_ = 0.598, *p* = 0.459, *η_p_*^2^ = 0.062] (Figure 4A). In contrast, for the ratio of ulnar NCV to ulnar nCSA, a significant main effect of sex was observed [*F*_(1,18)_ = 23.710, *p* = 0.001, *η_p_*^2^ = 0.748]. No effect of lateral preference was found [*F*_(1,18)_ = 0.480, *p* = 0.508, *η_p_*^2^ = 0.057], nor was there a significant interaction [*F*_(1,18)_ = 1.402, *p* = 0.270, *η_p_*^2^ = 0.149] (Figure 4B). Post hoc Tukey’s tests revealed a significantly greater ratio of ulnar NCV to ulnar nCSA for females compared to males in both the dominant (*p* = 0.022) and non-dominant arms (*p* = 0.022).

For the ratio of tibial nCSA to lower limb CSA, no significant main effects were found, with sex [*F*_(1,18)_ = 1.681, *p* = 0.227, *η_p_*^2^ = 0.157] and lateral preference [*F*_(1,18)_ = 2.518, *p* = 0.147, *η_p_*^2^ = 0.219]. Additionally, there were no significant interactions [*F*_(1,18)_ = 0.015, *p* = 0.906, *η_p_*^2^ = 0.002] (Figure 4C). Lastly. for the ratio of tibial NCV to tibial nCSA, no significant main effects were observed, with sex [*F*_(1,18)_ = 1.424, *p* = 0.267, *η_p_*^2^ = 0.151] and lateral preference [*F*_(1,18)_ = 0.111, *p* = 0.748, *η_p_*^2^ = 0.014] and no significant interactions were found [*F*_(1,18)_ = 0.215, *p* = 0.655, *η_p_*^2^ = 0.026] (Figure 4D).

**Figure 5:**
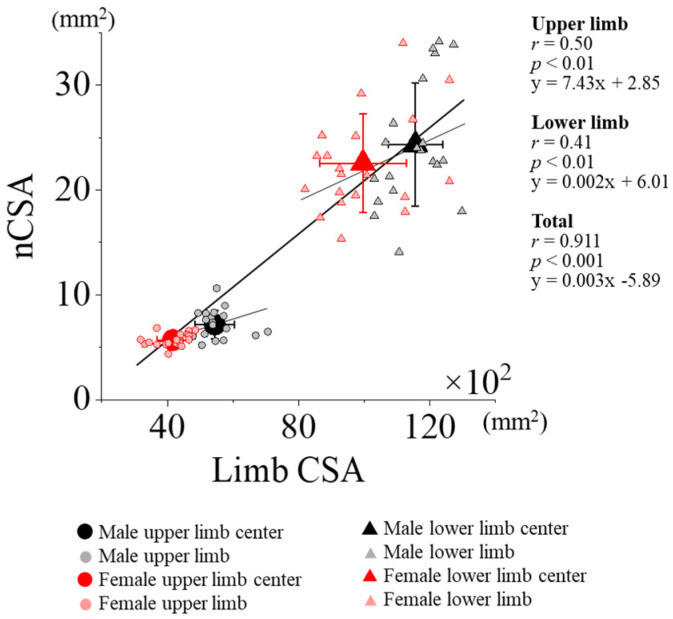
Relationship between nCSA and limb CSA for upper (circles) and lower (triangles) limbs in males (black) and females (red). The graph plots the limb CSA estimated from the circumference of each upper and lower limb against the corresponding nCSA. Individual data points are represented by small symbols, while larger symbols indicate mean values for each sex group.

Figure 5 shows the correlation between normalized nCSA and calculated limb CSA for both sexes. A strong, positive linear relationship was found across all data points (*r* = 0.91, *p* < 0.001). This indicates that nCSA remains relatively constant in proportion to limb CSA for both males and females.

## 4. Discussion

The purpose of this study was to examine sex differences in human NCV and nCSA between the upper and lower limbs. Our results revealed three main findings: (1) No sex differences exist in the ratio of nCSA to limb circumference in either the upper or lower limbs. (2) In the upper limbs, the absolute nCSA was greater in males than in females, irrespective of arm dominance, while NCV was higher in females than in males, also regardless of arm dominance. (3) In the lower limbs, no sex differences were observed in either nCSA or NCV. These findings indicate that nCSA is proportional to limb circumference without sex-specific differences. Additionally, the higher NCV in the upper limbs of females, not attributable to nCSA or forearm circumference, suggests that sex-specific factors may influence NCV. Conversely, the absence of sex differences in the lower limbs may relate to adaptations linked to human bipedalism. This discrepancy between the upper and lower limbs indicates the presence of plasticity in NCV and suggests that both intrinsic and extrinsic factors may influence sex-specific differences in peripheral nerve characteristics.

### 4.1. Relationship Between nCSA and Limb Circumference

Previous studies, e.g., Ref [26], have reported sex differences in peripheral nerve morphology. The present study confirmed these differences in terms of the absolute nCSA in the upper limbs. However, the ratio of nCSA to limb circumference was consistent between sexes, suggesting that the morphological development of peripheral nerves is related to muscle development. This implies that, similar to muscle, the influence of steroid hormone secretion plays a fundamental role in the development of peripheral nerves [15,27]. This relationship may ensure that nerve size scales appropriately with muscle mass. Interestingly, the nCSA to limb circumference ratio varied between the upper and lower limbs, regardless of sex. The present study does not explain the mechanism behind these limb-specific differences. Further research is needed to confirm whether this pattern exists in other human peripheral nerves and to elucidate the underlying mechanisms.

### 4.2. Potential Considerations of Height-Adjusted NCV and nCSA Values

Previous studies [7,8] have shown a relationship between height and NCV. It is important to note that the observed sex differences in the present study may be influenced by height. Re-examining the normalized NCV and nCSA values while accounting for height (see Table 2) revealed a trend similar to that found when height was not considered. These suggest that factors beyond body size contribute to the observed sex differences. These findings align with previous studies [17,18] showing no significant relationship between height and nCSA. Moreover, our findings indicate that other factors, in addition to height, may also play a role in NCV. This suggests that the sex differences identified in our study should not be attributed solely to variations in height. Therefore, further research is necessary to control height when studying both sexes.

### 4.3. Potential Mechanisms Underlying Sex Differences in Upper Limb nCSA and NCV

The observed differences in upper limb peripheral nerve characteristics between sexes likely result from a combination of biological and functional factors. Biologically, sex hormones may significantly influence myelin formation and ion channel function. Notably, estrogen has been shown to enhance myelin protein expression and alter sodium channel dynamics [27,28]. Previous studies have reported sex differences in fiber diameter distributions [26] and the impact of sex hormones on myelin formation and maintenance [29,30]. These hormonal influences may explain why females exhibit higher NCV despite having smaller nCSA.

The differential expression of sex differences between upper and lower limbs suggests distinct patterns of neuromuscular organization. Both upper and lower limbs experience functional demands and adaptations, but their evolutionary trajectories and functional requirements differ substantially. Upper limbs have evolved with greater flexibility in their functional roles, potentially allowing for a more pronounced expression of sex-specific characteristics. Lower limbs, in contrast, have experienced selective pressure for bipedal locomotion, which may have favored similar neuromuscular properties in both sexes. This interpretation is supported by studies showing consistent patterns of muscle activation and force production in lower limbs across sexes during locomotion [31].

Furthermore, the relationship between nCSA and limb circumference reveals an intrinsic scaling principle in peripheral nerve development. This principle appears to be conserved across sexes, suggesting that while absolute nerve sizes may differ, their relative proportions maintain a stable connection with the mass of the innervated tissue. This finding has important implications for our understanding of both developmental biology and clinical assessment methods.

### 4.4. Absence of Sex Differences in Lower Limb nCSA and NCV

During development, the lower limbs of both sexes experience similar biomechanical loads, likely due to the fundamental requirements of bipedal locomotion. This observation aligns with studies showing that bone and muscle development in the lower limbs demonstrates greater morphological similarity between sexes than in the upper limbs [32]. Similarly, peripheral nerve development may exhibit region-specific patterns, which could explain the differences in nerve characteristics between upper and lower limbs. The evolution of obligate bipedalism, a uniquely human characteristic, appears to have shaped lower limb neuromuscular function differently from upper limbs [31]. The evolutionary pressure for efficient bipedal locomotion has likely led to a convergence of lower limb nerve characteristics between males and females, effectively overriding the inherent sex-specific differences observed in the upper limbs.

### 4.5. Methodological Considerations and Clinical Implications

While our study provides valuable insights, several limitations should be acknowledged: (a) A key limitation of this study is the relatively small sample size (*n* = 20). While we detected significant differences in our primary outcomes, some subtle effects might have gone unnoticed. Future studies with larger sample sizes would be valuable to confirm our findings and potentially identify additional sex-specific characteristics in peripheral nerve properties. (b) The current in vivo human imaging technology, with a resolution limit of approximately 50 μm, cannot identify individual axon diameters. Our measurements of nCSA, while informative, do not capture finer details of nerve fiber structure. Future research should employ high-resolution imaging studies, potentially combining advanced ultrasonography with deep learning image processing, to explore these finer details of nerve structure. (c) The present study included healthy adults within a specific age range. NCV changes with development and aging [33,34] and is also affected by neurological disorders [24,35]. To comprehensively demonstrate the sex specificity of NCV, future studies should investigate sex differences across various age groups and in individuals with different health conditions. Also, the potential influence of height on nerve characteristics represents a methodological consideration that warrants further investigation. Future studies should consider systematic participant selection with matched height groups to better elucidate the relationship between body size and peripheral nerve properties while accounting for the complex anatomical and physiological variations that occur along the length of peripheral nerves. (d) If sex specificity is due to acquired factors, longitudinal studies tracking NCV from childhood through adulthood, as well as cross-cultural comparisons, could provide further insights into the nature of these sex differences. (e) The present findings are specific to the ulnar and tibial nerves. Given that sex differences in muscle mass and strength primarily occur in the upper limbs and not in the lower limbs [36], additional research is necessary to determine whether similar sex differences exist in other peripheral nerves. (f) The findings suggest that sex-specific reference values for NCV may be necessary, particularly for upper limb nerves. Establishing these reference values could improve the accuracy of diagnosing peripheral neuropathies and monitoring treatment efficacy.

## 5. Conclusions

This study provides evidence of sex-specific differences in upper limb NCV and nCSA. Our findings demonstrated that females exhibit higher NCV despite having smaller nCSAs, specifically in the upper limbs, while no such differences were observed in lower limb nerves. This observation challenges existing assumptions about the relationship between nerve size and conduction velocity. These findings emphasize the need for sex-specific consideration in upper limb neurophysiological assessments. The results have significant implications for both the diagnosis and treatment of peripheral neuropathies, highlighting the importance of establishing sex-specific reference values for nerve conduction studies of upper limb nerves. Future research should focus on elucidating the underlying mechanisms behind these sex differences, which could lead to more personalized approaches in neurology and rehabilitation medicine.

## Figures and Tables

**Table 1:** Physiological characteristics for male and female groups.

Group	Age (Years)	Height (cm)	Body Mass (kg)	Forearm Length (mm)		Lower Leg Length (mm)	
				Dominant Arm	Non-Dominant Arm	Supporting Leg	Reacting Leg
Male (*n* = 10)	20.3 ± 2.2	175.1 ± 7.6	72.7 ± 8.5	247 ± 18.6	246 ± 18.3	364 ± 21.4	363 ± 21.0
Female (*n* = 10)	20.1 ± 1.0	160.7 ± 6.0 *	55.9 ± 6.0 *	222 ± 11.8 *	220 ± 10.8 *	333 ± 17.6 *	333 ± 17.0 *

Values are expressed as means ± SD. * Shows significant differences between males and females (*p* < 0.05).

**Table 2:** Sex differences in height-adjusted NCV and nCSA values.

Group	Δ NCV of Ulnar Nerve/Height	Δ NCV of Tibial Nerve/Height	Δ nCSA of Ulnar Nerve/Height	Δ nCSA of Tibial Nerve/Height
Male (*n* = 10)	0.308 ± 0.037	0.344 ± 0.064	0.041 ± 0.006 *	0.139 ± 0.029
Female (*n* = 10)	0.348 ± 0.055	0.358 ± 0.037	0.035 ± 0.002	0.140 ± 0.030

Values are expressed as means ± SD. * Shows significant differences between males and females (*p* < 0.05).

## Data Availability

The original contributions presented in the study are included in the article; further inquiries can be directed to the corresponding authors.
